# Efficacy and toxicity of hydroxyurea in mast cell activation syndrome patients refractory to standard medical therapy: retrospective case series

**DOI:** 10.1007/s00210-022-02282-8

**Published:** 2022-08-19

**Authors:** Leonard B. Weinstock, Jill B. Brook, Gerhard J. Molderings

**Affiliations:** 1 Clinical Medicine, Department of Medicine, Washington University School of Medicine, President, Specialists in Gastroenterology, 11525 Olde Cabin Road, St. Louis, MO 63141 USA; 2 Truckee, USA; 3 Institute of Human Genetics, University Hospital Bonn, 53127 Bonn, Germany

**Keywords:** Mast cell activation syndrome, Treatment, Hydroxyurea, Efficacy, Toxicity

## Abstract

Determine efficacy and adverse events (AEs) of hydroxyurea (HU) in mast cell activation syndrome (MCAS) patients who were refractory to standard medical therapy. An electronic chart review was performed to find MCAS patients who received HU in a MCAS medical practice. Diagnosis of MCAS was established on the basis of mast cell (MC) activation symptoms in ≥ 5 systems plus ≥ 1 abnormal MC mediators and/or ≥ 20 MC/high power field on duodenal biopsies. Medicines not providing significant clinical improvement prior to HU were tabulated. The following symptoms were evaluated by patients on a 0–10 scale prior to and at the study conclusion: bone pain, abdominal pain, diarrhea, bloating, and nausea. Safety labs were obtained on a regular basis. Twenty out of three hundred ten (8.4%) MCAS patients received HU. Patients included 22 females, average age 42.4 years. Dysautonomia was present in 60%. An average of 10.6 (SD 1.7, range 8–13) medications were used prior to adding HU to various concomitant medications. Average dose of HU was 634 mg. In 20 patients who continued therapy for ≥ 2 months, there was statistically significant reduction of bone pain, abdominal pain, diarrhea, bloating, and nausea. Fourteen patients noted prolonged success with therapy. Six patients stopped HU within 6 weeks owing to AEs. Four patients treated ≥ 2 months had AEs and 2 led to HU cessation. All AEs were reversible. Refractory MCAS patients showed clear significant improvement in bone pain and gastrointestinal symptoms on HU. Systematic monitoring was effective in preventing the occurrence of severe HU-induced adverse events.

**Supplementary Information:**

The online version contains supplementary material available at 10.1007/s00210-022-02282-8.

## Introduction

Mast cell activation syndrome (MCAS) is the most common variant of mast cell activation disease (MCAD) and causes chronic inflammatory and allergic symptoms and syndromes (Hamilton 2018; Afrin et al. 2017). In a German study, the prevalence of MCAD was 17% (Molderings et al. 2013). The pathologic behavior of mast cells (MCs) in MCAS is due to uncontrolled activation and release of mediators by aberrant MCs leading to harmful local and distant effects (Frieri 2018; Ravanbakhsh and Kesavan 2019). This may be due to mutations of the mast cell (MC) regulatory genes (Molderings et al. 2007; Molderings 2015). MCAS, in its wide variety of clinical presentations, features inappropriate MC activation with relatively modest MC proliferation (in contrast to systemic mastocytosis (SM), the rare variant of MCAD).

Establishing the diagnosis of MCAS is paramount. Although being highly prevalent, this syndrome is generally not considered in the differential diagnosis of a multisystemic disorder (Afrin et al. 2016). Diagnosis may be difficult to prove but is important because the disease is treatable (Afrin et al. 2020). In a study of 413 MCAS patients, symptoms that were present in 50% or more patients were, in descending frequency, fatigue, abdominal pain, altered bowel habits, muscle pain, pre-syncope or syncope, headaches, itching, urticaria, nausea, chills, edema, eye irritation, dyspnea, and heartburn (Afrin et al. 2017). Unrecognized and untreated MCAS may account for refractory gastrointestinal (GI) symptoms which is often attributed to functional GI disorders including irritable bowel syndrome (Hsieh 2018; Weinstock et al. 2021). Among the common often severe symptoms affecting all body systems, migratory bone pain is a particularly severe clinical phenomenon (Afrin 2013). In the differential diagnosis of migratory bone pain, MCAS is not considered a potential etiology (Mantyh 2019). In light of the migratory nature of bone pain in MCAS, it is possible that release of mediators locally or distant leads to inflammation of the periosteal nerves leading to pain (Mach et al. 2002). Bone pain in MCAS patients responds poorly to narcotics, non-narcotic analgesics, antidepressants, and anticonvulsants (Afrin 2013).

There are no FDA-approved or EU-approved medications specifically for MCAS, treatment can be difficult in a sizable number of patients, and, thus, alternative medical therapy is important to investigate. Hydroxyurea (HU) is an oral ribonucleotide reductase inhibitor with antimetabolic and antineoplastic properties (Musiałek and Rybaczek 2021). It inhibits ribonucleotide reductase, a ubiquitous intracellular enzyme that converts ribonucleotides to deoxyribonucleotides, which are required for DNA synthesis and repair. First used 60 years ago for chronic myeloproliferative neoplasms, HU is now standard of care in reducing the severity of sickle cell disease (SCD) (McGann and Ware 2015). In this context, it is of interest that in animal models and human subjects with SCD, medications that reduce MC activation decreased bone pain (Vincent et al. 2013, 2016). HU also inhibits replication of human immunodeficiency virus-1 (HIV-1) and has been used in therapy of cyanotic congenital heart disease (Lori and Lisziewicz 2000; Reiss et al. 2007). A theoretic explanation for effectiveness of HU in these diseases include reducing MC activity independent of anti-proliferative effects. HU could decrease activity and ability of GI mucosal MCs that capture HIV-1 and mediate trans-infection of CD4 + T cells (Jiang et al. 2015). Similarly, HU could reduce the activity of the increased numbers of chymase-containing MCs in lung tissue of congenital heart disease patients and, thereby, decrease severity of these pulmonary and cardiac diseases (Hamada et al. 1999).

HU has also been used in the treatment of systemic MC activation disease (Afrin 2013, 2014). It achieved modest benefit in SM with an associated hematological neoplasm (Lim et al. 2009). Five MCAS patients treated with HU had marked reduction in diffuse body and bone pain (Afrin 2013). In that study, cytopenia needed cessation of the drug in one patient. Reactions to excipients in three patients required trials of an alternative formulation which were then successful.

The aim of the present retrospective analysis is to address the effectiveness and toxicity of HU in MCAS patients who are refractory to standard medical treatment options.

## Methods

The study was reviewed by the Sterling Investigational Review Board (IRB) in Atlanta, Georgia. The Sterling IRB Chairperson decided that the study (ID #9768) was exempt from full IRB review pursuant to the terms of the U.S. Department of Health and Human Service’s Policy for Protection of Human Research Subjects at 45 C.F.R. §46.104(d). The IRB found that the exemption category 2 applied. All patients signed an informed consent form that described all risks of HU and allowed collection and reporting of clinical data.

One of the investigators (LBW) is an internist and gastroenterologist specializing in MCAS patients. An electronic chart review search of his patient data was performed to find MCAS patients who were prescribed HU. These patients had failed to show any significant clinical response to step 1, 2, 3, and/or 4 MC medicines plus elimination dietary trials as per recent guidelines (Weinstock et al. 2021). A variety of other medications reported to be effective in MCAS were employed including benzodiazepines, tricyclic antidepressants, imatinib, and low-dose naltrexone (Molderings et al. 2016; Weinstock et al. 2018; Weinstock and Blasingame 2020).

The diagnosis of MCAS was established on the basis of the diagnostic Consensus-2 criteria (Afrin et al. 2020), in particular on the constellation of complaints attributable to pathologically increased MC activity in ≥ 5 systems plus either ≥ 1 minor criteria, i.e., abnormal MC mediators and/or ≥ 20 MC/high power field on duodenal biopsies. The standardized validated mast cell mediator release syndrome (MCMRS) checklist was used to determine the number of MC symptoms and systems for each patient (Appendix 1). The total score considers the points for the number of symptoms, abnormal MC mediators, and abnormal MC count on biopsy (Molderings et al. 2013; Weinstock et al. 2021). At a score of ≥ 14 points, the presence of a MCMRS has a probability of 95%. We also used a MCMRS score for symptoms alone. Prior medicines that did not give significant clinical improvement and concomitant medications were tabulated. The following symptoms were evaluated by a 0 to 10 scale prior to HU therapy: bone pain, abdominal pain, diarrhea, bloating, and nausea. The baseline symptoms were derived from the intake MCMRS checklist administered to all new complex patients seen in the clinic. These symptoms were determined at a second point in time at the date of study commencement in November 2021. HU was prescribed either as the generic form at 500 mg daily or as branded Droxia (Bristol-Meyers Squibb Company, Princeton, NJ) 600 mg daily Droxia directions were 200 mg daily and increase the dose by 200 mg every three days until 600 mg was reached. The choice was dependent on insurance coverage. Dose escalation beyond 500 or 600 mg was considered on a case-by-case basis for non-responders.

Safety lab monitoring included a baseline complete blood count (CBC) and complete metabolic profile (CMP). The CBC was obtained weekly for the first month, biweekly for the second month, and then monthly if stable. The CMP was performed monthly. Patients were encouraged to contact the clinician for any adverse events and were seen in the clinic at 3-month intervals. Statistical tests for symptom score changes were performed using R software and statistical significance was defined as *p* < 0.05. Repeated measure *t*-tests were used to decide whether symptoms significantly improved after treatment with HU.

## Results

Of 310 MCAS patients, 26 (8.5%) were prescribed HU. There were 22 females and 4 males with an average age of 42.4 years. In the twenty-six patients, the mean MCMRS symptom score was 24. Co-morbid syndromes included postural orthostatic tachycardia syndrome (POTS) in 61.5% and hypermobile Ehlers-Danlos syndrome in 34.6%. An average of 10.6 (SD 1.7, range 8–13) medications were prescribed prior to adding HU to an average of 6.0 concomitant medications. The duration of HU therapy was < 2 months in 6 patients and an average of 15 months (SD 12, range 2–42) in the other 20 patients. Table 1 summarizes the clinical characteristics, medication history, and adverse events.

**Table 1:** Clinical characteristics, medicine history, and adverse events for twenty-six MCAS patients treated with hydroxyurea (HU)

Females, *N* (%)	22 (84.6%)
Males, *N* (%)	4 (15.4%)
Age, mean (SD)	42.4 (14.3)
MCMRS score, mean (SD)^a^	24.2 (4.2)
Percentage of patients with abnormal mast cell mediator level(s)	70%
Percentage of patients with positive biopsy^b^	95%
Postural orthostatic tachycardia syndrome, *N* (%)	16 (61.5%)
Hypermobile Ehlers-Danlos syndrome, *N* (%)	9 (34.6%)
Number of medications used prior to starting HU, mean (SD, range)	10.6 (1.7, 8–13)
Number concomitant medications taken with HU, mean (SD, range)	6.0 (2.3, 1–9)
HU dose, mean (SD)	634.6 (135.5)
Duration of HU therapy, mo, mean (SD)	9.9 (11.1)
Adverse events, *N* (%)	10 (38.5%)
Cessation due to adverse events, *N* (%)^c^	8 (30.8%)

^a^The mast cell mediator release syndrome (MCMRS) score was calculated by tabulating a standardized validated checklist. A score of ≥ 14 is highly suggestive of a MCMRS. ^b^The immunohistochemistry CD117 stain was performed in duodenal biopsies in 20 patients. In the patient with a normal biopsy, the diagnostic minor criterion of an increased mediator release was positive. ^c^Adverse events leading to cessation of therapy in 8 patients included: mild changes in the leukocyte count (1), creatinine (2), and liver enzymes (1) in 4 patients; and worsening of chronic symptoms of poor healing in 1 patient; and a variety of symptoms including bone pain, nausea, headaches, and fatigue in 3 patients

Medications used prior to HU are delineated in Table 2. Initial therapy usually was a combination of histamine-1 and -2 receptor antagonists, quercetin, vitamins C and D, and low-dose naltrexone. Then, one or more of the following drugs were added or substituted by another one: cromolyn, oral ketotifen, montelukast, aspirin, tricyclic antidepressants, and benzodiazepines. Invariably blockade of both histamine receptor types were continued throughout the course of therapy. Omalizumab was used concomitantly in eight patients—it often improved urticaria, but did not affect other MCAS symptoms. Other advanced medications that did not help prior to starting HU included zileuton (in seven patients), imatinib (in two patients), and intravenous immune globulin (in two patients). Short courses of budesonide or prednisone were prescribed to five patients.

**Table 2:** Medications that failed to provide significant improvement in those twenty mast cell activation syndrome patients who for that reason were treated with hydroxyurea for ≥ 2 months

Medication	N	%
Histamine H1 receptor antagonist	20	100
Histamine H2 receptor antagonist	19	95
Cromolyn	19	95
Vitamin C	19	95
Montelukast	18	90
Low-dose naltrexone	17	85
Quercetin	16	80
Vitamin D	15	75
Benzodiazepine	12	60
Ketotifen	11	55
Aceytsalicylic acid	9	45
Omalizumab	8	40
Zileuton	7	35
Norepinephrine/5-hydroxytryptamine enhancers	6	30
Glucocorticoid receptor agonist ^a^	5	25
Imatinib	2	10
Intravenous immunoglobulin^b^	2	10
Pentosan polysufate	1	1

^a^Glucocorticoid receptor agonist used included budesonide and short courses of prednisone

^b^Intravenous immunoglobulin was used for severe concomitant dysautonomia

**Fig. 1:**
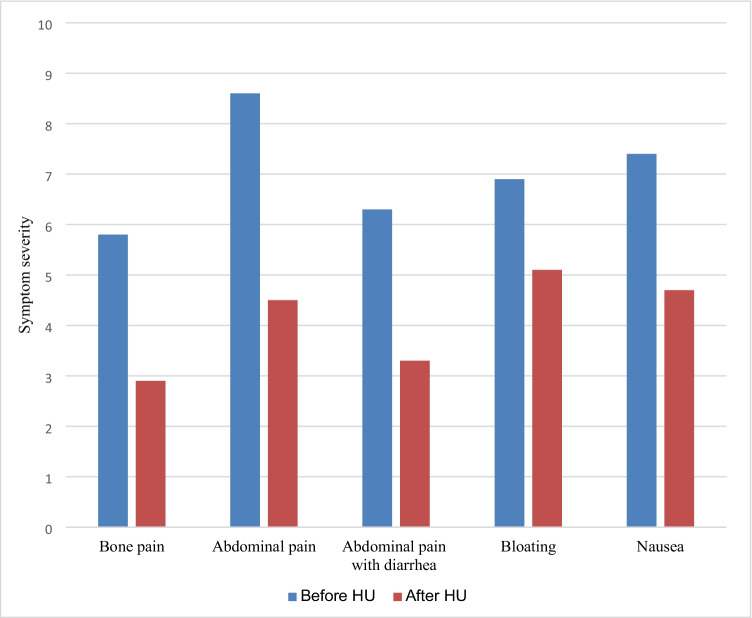
Outcome of twenty MCAS patients treated with hydroxyurea (HU) ≥ 2 months (mean 15 ± 12; range 2–42). The differences in the symptom severity before and after hydroxyurea treatment were at least significant at < 0.01, *t*(19), *t* tests with 19 degrees of freedom

The average dose of HU was 634 mg (SD 135, range 500–1000) which was administered for 9.9 months (SD 11.1, range 0.5–42.0) in all twenty-six patients (Table 1). The analysis for the 20 patients who took HU ≥ 2 months showed overall statistically significant benefit in all of the symptom domains (Table 3, Fig. 1). Fourteen of these twenty patients (70%) had significant benefit and were considered to be “long-term users”: 32.6 months (SD 11.8, range 4–42). One patient needed a change in formulation due to hives from an excipient. Five patients took HU ≥ 2 months but did not achieve significant clinical response and one of patient had worsening symptoms. One patient had an excellent response for 12 months but stopped HU owing to a self-limited increase in creatinine. To decide whether there was a difference in response to HU as determined by the presence of bone pain, we separated the patient group by presence or absence of bone pain (*N* = 15 vs. *N* = 5) and compared symptom response of abdominal pain, diarrhea, bloat, and nausea between both groups. Improvement scores were not statistically different between those with and without bone pain.

**Table 3:** Symptoms rated on a scale of 0 to 10 before and after hydroxyurea (HU) treatment in twenty MCAS patients who took HU ≥ 2 months

Symptom	Mean (SD) severity before HU	Mean (SD) severity after HU	*t*(19) =	*p* value
Bone pain	5.8 (4.0)	2.9 (3.4)	3.0	0.0066
Abdominal pain	8.6 (1.8)	4.5 (2.7)	6.0	< 0.0001
Diarrhea	6.3 (3.9)	3.3 (3.6)	4.2	0.0005
Bloating	6.9 (3.4)	5.1 (3.1)	3.3	0.0040
Nausea	7.4 (3.3)	4.7 (3.2)	3.9	0.0008

Abbreviations: *SD*, standard deviation; *t(19)*, *t* tests with 19 degrees of freedom

Self-limited adverse events led to cessation of therapy within 2 months in six patients. Mild changes in the leukocyte count, creatinine, and/or liver enzymes were detected in three patients. There was worsening of poor healing of skin wounds in one patient who suffered from IgG deficiency; bone pain in one patient; and nausea/headaches/fatigue in one patient. Two of the twenty other patients had self-limited adverse events that led to cessation of HU: 1) one patient who had complaints of fatigue, edema, and dizziness and subsequently improved with imatinib and (2) one patient with transient increased creatinine. Two long-term patients had self-limited adverse events but were able to continue HU therapy: (1) one patient had a transient increased liver enzyme (γGT) due to intake of high dose acetaminophen during a COVID-19 infection and (2) one patient with mild leukopenia resolved by dose reduction. One patient had urticaria in response to taking multiple medicines. She had hives with two forms of hydroxyurea but found a third that did not have a dye that she reacted to.

## Discussion

In the present retrospective study, we addressed the effectiveness and toxicity of HU in MCAS patients who were refractory to conventional treatment options. Twenty-six patients did not respond to dietary changes and an average of 10.6 medicines and 6.0 concomitant medicines before adding HU to the regimen. The prevalence of postural orthostatic tachycardia and hypermobile Ehlers-Danlos syndromes were higher in this cohort than in other case series of MCAS patients (Afrin et al. 2017; Weinstock and Blasingame 2020). These MCAS patients are considered to be more difficult to treat and there is an overlap of symptoms in the three syndromes (Weinstock et al. 2021). Overall, 14/20 (70%) patients who took HU ≥ 2 months experienced significant clinical benefit from HU and continued it long-term (average of 32.6 months).

AEs were reversible with drug cessation or decreasing the dose. Laboratory changes were all mild and included leukopenia (1), neutropenia (1), liver enzyme changes (2), and increased creatinine (2). The safety laboratory plan employed was effective in detecting the above problems before clinical symptoms arose. This is important because when using HU long-term the possibility of developing severe adverse effects must be kept in mind. HU may cause severe myelosuppression (Agrawal et al. 2014). Therefore, it should not be prescribed if the bone marrow function is depressed. HU has been used primarily for the treatment of myeloproliferative diseases, which has an inherent risk of transforming to acute myeloid leukemia. There has been a longstanding concern that HU itself carries a leukemia risk, but large studies have shown that the risk of secondary malignancy is absent (Szikriszt et al. 2016; Santoro et al. 2017). In patients with essential thrombocythemia treated with HU, the rate of secondary malignancy was 7% and, hence, not different from those patients without any therapy (5%) (Santoro et al. 2017). Concerns about the secondary malignancy risk from HU, originally thought to be fairly high, have been diminishing over the decades, to the point where long-term HU use is now standard of care in certain subpopulations of polycythemia vera patients and in many sickle cell patients. In fact, there are many studies now demonstrating modest to no increased risk for malignancies from long-term HU use (e.g., Rodriguez et al. 2018; Tolu et al. 2020; Liggett et al. 2022). Even among the studies suggesting an increased risk of “skin cancers” in long-term HU users, it is critical to note that the observed excess of cutaneous cancers were non-melanomatous cancers, with far different prognosis than melanomas, the development of which long-term HU use does not seem to drive (Soutou et al. 2020). Nevertheless, patients should use sun protection with UV blocking cream. Liver and renal toxicity was observed in one study (Lisziewicz et al. 2003). HU pulmonary toxicity is a rare complication (Sandhu et al. 2000). There may be potential risk to a fetus and mothers who are breast feeding should not take HU (Stevens 1999). In men with SCD, a risk of reduced fertility after HU therapy has been reported (DeBaun 2014).

The main limitations of the study are the retrospective and open label design. Another issue is that symptom assessments were conducted only at the date of entry into the clinic and at the time of data collection at the end of the study. The patients who stopped taking HU earlier than the end of data collection completed the symptom questionnaire in a retrospective manner. The possibility of recall bias cannot be excluded in this study. Finally, after step-1 therapy failed to be effective, the subsequent replacements and concomitant medicines were not prescribed in a regimented manner so that such medicine changes could have contributed to the HU-induced improvement.

In conclusion, refractory MCAS patients showed clear statistically significant improvement of bone pain and gastrointestinal symptoms by HU. Systematic monitoring was effective in detecting minor laboratory changes.

## Supplementary Information

Below is the link to the electronic supplementary material.

Supplementary file1 (DOC 270 KB)

Supplementary file2 (XLSX 88 KB)

## Data Availability

The raw data are given in the supplementary file ESM_2-xlsx.
