# Supplementary material for: Efficacy and toxicity of hydroxyurea in mast cell activation syndrome patients refractory to standard medical therapy: retrospective case series
Source: Naunyn Schmiedebergs Arch Pharmacol. 2022 Aug 19;395(11):1441–7. doi: 10.1007/s00210-022-02282-8 (PMC9388361; doi:10.1007/s00210-022-02282-8)
Supplement: Supplementary file 1 — Supplementary file1 (DOC 270 KB) [file 210_2022_2282_MOESM1_ESM.doc]

**Efficacy and Toxicity of Hydroxyurea in Mast Cell Activation Syndrome Patients Refractory to Standard Medical Therapy: Retrospective Case Series**

Naunyn-Schmiedeberg´s Archives of Pharmacology

Leonard B. Weinstock, Jill B. Brook, Gerhard J. Molderings

**Corresponding author:** Leonard B. Weinstock, Department of Medicine, Washington University School of Medicine, MO, [lw@gidoctor.net](mailto:lw@gidoctor.net)

Caption: **Standardized validated *Mast Cell Mediator Release Syndrome Questionnaire***

**Mast Cell Mediator Release Syndrome Questionnaire**

Patient name ________________________ Date ______________

Date of birth _______________

**Answer all of the following symptoms/questions, even if they are only slightly bothersome, rarely occurring (for instance, not necessary present currently but in the past), or may seem not be related to your main problems.** Contact your doctor if you have difficulty completing the questionnaire.

Check (✓) inside the box if the statement applies to you.

If the statement applies to you, enter the intensity level when it was present the last time it occurred on the line next to the box. Please use the range of 1 (very mild) to 10 (unbearable) to reflect the level of your discomfort.

1 2 3 4 5 6 7 8 9 10

**CONSTITUTIONAL** **Applies Intensity**

Significant physical weakness or fatigue doing everyday activities □ **1** ___

Extreme fatigue attacks, so it is hard to keep eyes open □ **1** ___

At times I lose weight despite maintaining my normal diet □ **1** ___

Complaints arise or existing complaints are worsened by:

Sleep deprivation (awake for more than 24 hours)...... □ **1** ___

Hunger or fasting (no food all day).............................. □ **1** ___

High histamine foods (such as red wine, cheese, chocolate, tuna, cured fish/meat, left-over meat)……………….. □ **1** ___

Alcohol consumption.................................................. □ **0** ___

Physical exertion......................................................... □ **0** ___

Heat............................................................................. □ **0** ___ Cold............................................................................. □ **0** ___

Stress…....................................................................... □ **0** ___

**EYES/EARS/NOSE/MOUTH**

The following occur repeatedly or may be constant:

Ears have ringing or odd sounds and/or □ ___ Eyes are dry, itchy, red, burning, or feel gritty and/or □ ___

Runny nose or stuffy nose and/or □ ___

Inflammation or ulcers of the mouth □ ___

Score 1 if one or more is present. □ **1**

**CHEST and HEART**

The following occur repeatedly or may be constant:

Burning and/or pressure pain in the chest and the heart tests were normal (electrocardiogram and/or stress test) □ **1** ___

Rapid heart rate (palpitations) □ **1** ___

Redness or flushing of the skin, especially face or upper body □ **2** ___

Hot flashes (these usually last 2 to 5 minutes and rarely 10 minutes and are often accompanied by nausea or other symptoms; these are not

hot flashes of menopause) □ **2** ___

Sudden dizziness/lightheadedness with fainting or near faint □ ___

Sudden temporary increase in blood pressure □ ___

Score 2 if one or more is present. □ **2**

I have seen evidence for pulse and blood pressure changes using my □ digital watch device

**LUNGS**

The following occur repeatedly or may be constant:

Irritable dry cough or need to cough and/or □ ___

Feeling of shortness of breath or difficulty taking a full breath and/or □ ___

Asthma-like complaints (wheezing) □ ___

Score 1 if one or more is present. □ **1**

**ABDOMEN**

The following occur repeatedly or may be constant:

Nausea (with or without vomiting) □ **1** ___

Pain in the abdomen □ **1** ___

Character of pain: burning □ **1** ___ Character of pain: crampy or spastic □ **1**  ___

Character of pain: it is associated with diarrhea □ **1** ___

Marked attacks of visible bloating or distension within minutes (up to around 10 minutes) □ **1** ___

A surgeon told me that adhesions (scar tissue) were seen during my very first laparoscopy or abdominal/pelvic surgery □

**URINE/PELVIS**

The following occur repeatedly or may be constant:

Bladder and/or pelvic pain (this applies to women and men) and is often associated with painful, frequent and/or urgent urination

and may be associated with pain during sex. □ **1** ___

During these times bacterial cultures and urine analysis are normal. □

I have had these symptoms but have not seen a doctor to order tests. □

**NEUROLOGIC**

The following occur repeatedly or may be constant:

Headaches (may be throbbing on one side only or have previously been diagnosed as a migraine) □ **1** ___

Brain fog – word finding problems and/or concentration difficulties

with or without associated insomnia episodes. □ **1** ___

Neuropathy: leg pain or arm pain and/or altered feelings (numbness, tingling, pins and needles). This does not respond to over-the-counter pain medicine. □ **1** ___

**SKIN – see last page for photograph examples**

The following occur repeatedly or may be constant:

Hives (red raised itchy spots) □ **1** ___

Itching with or without skin changes □ **0** ___

Itchy skin lesions that look like acne in the corners of the nasal-lip area, as well as, the chin and forehead during attacks □ **1** ___

Itching in area around the anus during attacks □ **1** ___

Painless, non-itchy swelling (especially lips, cheeks, eyelids) □ **1** ___

Reddish-brown spots and/or knots under the skin □ **2** ___

Hemangiomas ("blood sponges") □ **1** ___

**HEMATOLOGIC**

The following occur repeatedly or may be constant:

Bruising after minor injuries □ ___

and/or

Unusual nose bleeds □ ___

and/or

(Women with significantly increased menstrual bleeding) □ ___

Score 1 if one or more is present. □ **1**

**BONE**

Bone pain that usually occurs in more than one bone □ **1** ___

Bone density test showed osteoporosis or osteopenia

and/or □

Whole-body nuclear scintigraphy showed areas of increased

bone metabolism without a known cause □ 

Score 1 if one or both is/are present. □ **1**

**General Questions**

Do you get colds regularly which then turn into bacterial infections such as bronchitis or sinus infections? □ **1**

Has the course of your illness been episodic (and/or with attacks)? □ **1**

Have symptom-free periods become shorter? □ **1**

Any relief of nausea with ondansteron (example: Zofran) or antihistamines (examples: diphenylhydramine, lorantidine, cetirizine)? □ **1**

Do you know with relative certainty the beginning of your gastrointestinal and/or other complaints that is linked to a memorable event (infection, stress, environmental change, etc)? □

If yes, when and which events? __________________________________________

____________________________________________________________________

Have your parents, siblings and/or children had similar diseases or syndromes to yours (such as intestinal complaints, food intolerances, pulmonary complaints, allergies, migraine-like headache, pains in various systems without apparent cause, skin changes, hives, itching, runny nose, recurring eye irritation, ringing in the ears, tendency to bruise)? □

List these affected relatives: ______________________________________________

List of your medications, vitamins, and supplements used regularly or as needed:

____________________________________________________________________

____________________________________________________________________

____________________________________________________________________

____________________________________________________________________

Medicine allergies/reactions: ____________________________________________________________________

____________________________________________________________________

Food allergies/reactions: _________________________________________________

_____________________________________________________________________

Environmental reactions (odors, temperature, lights, etc.): ______________________

_____________________________________________________________________

Mold exposure: ________________________________________________________

Tick bite history: _______________________________________________________

Weight: ____kg (or ____pounds); Height: ____cm (or ___ feet and ___inches)

**SKIN PHOTOGRAPHS**

Hives Acne-like lesions


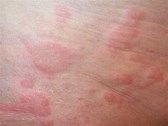

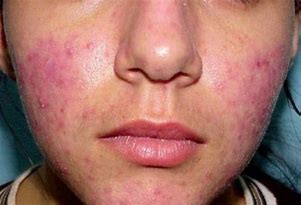


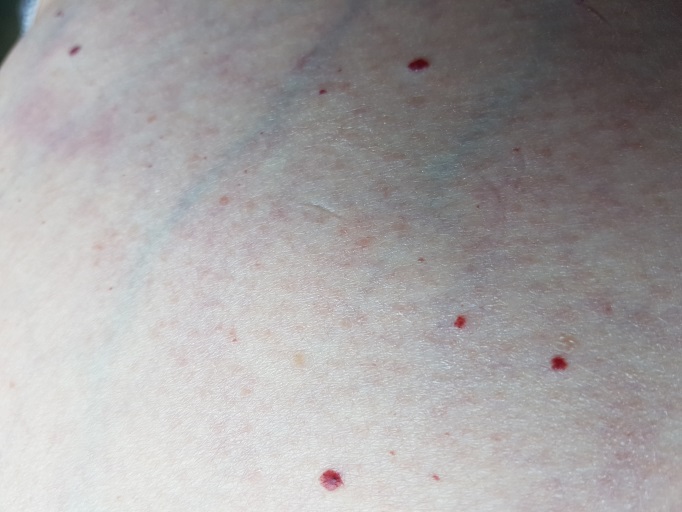
Reddish-brown spots Knots under skin Hemangiomas
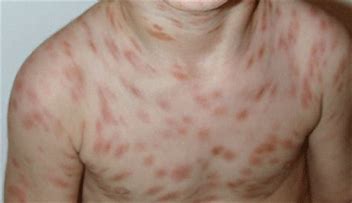

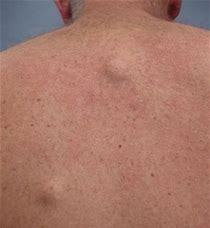


**Laboratory Data**

At least once during the disease phases there was:

**Applies**

Hyperbilirubinemia up to about 2.5 mg% with the exclusion of

Meulengracht/Gilbert’s syndrome or another hereditary disorders □

Increase in transaminases:

γGT and/or □

ALT and/or □

AST and/or  □

Score 1 if one or more is present. □ **1**

*AST increased >10 fold (subtract 1 point and look for other diseases) □ -****1***

Hypercholesterolemia (patient must be normal or underweight) □ **1**

Low titer autoantibodies without a corresponding organ symptom □ **1**

**Mast cell mediators:**

Tryptase in serum was normal □ **0**

Tryptase was marginally increased □ **3**

Tryptase increased >2 times the upper limit □ **10**

N-methylhistamine in urine was normal □ **0**

N-methylhistamine was marginally increased □ **1**

N-methylhistamine was 10 times the upper limit □ **5**

N-methylhistamine >10 times the normal limit □ **10**

Chromogranin-A in serum was normal □ **0**

Chromogranin-A was increased (and other causes were excluded) □ **3**

Heparin and/or factor VIII in plasma was/were normal □ **0**

Heparin and/or factor VIII was/were elevated (and bleeding disorders were excluded). □ **3**

Other conspicuous laboratory findings (please name with values) □ **0**

_________________________________________________________________________________

_________________________________________________________________________________
